# Determination of Vertical Interproximal Bone Loss Topography: Correlation Between Indirect Digital Radiographic Measurement and Clinical Measurement

**DOI:** 10.5812/iranjradiol.7732

**Published:** 2012-06-30

**Authors:** Farzad Esmaeli, Adileh Shirmohammadi, Masoumeh Faramarzie, Nader Abolfazli, Hossein Rasouli, Saied Fallahi

**Affiliations:** 1 Department of Oral and Maxillofacial Radiology, Faculty of Dentistry, Tabriz University of Medical Sciences, Tabriz, Iran; 2 Department of Periodontics, Faculty of Dentistry, Tabriz University of Medical Sciences, Tabriz, Iran

**Keywords:** Radiography, Dental, Bone Diseases, Topography, Medical

## Abstract

**Background:**

Diagnosis and accuracy in determining the exact location, extent and configuration of bony defects of the jaw are of utmost importance to determine prognosis, treatment planning and long-term preservation of teeth. If relatively accurate diagnosis can be established by radiography, proper treatment planning prior to treatment procedures will be possible.

**Objectives:**

The aim of the present study was to assess the correlation between indirect digital radiographic measurements and clinical measurements in determining the topography of interproximal bony defects.

**Patients and Methods:**

Twenty interproximal bony defects, preferably in the mandibular and maxillary 5↔5 area were selected and radiographed using the parallel periapical technique. The radiographs were corrected and digitized on a computer using “Linear Measurement” software; then the three parameters of the base of defect (BD), alveolar crest (AC) and cementoenamel junction (CEJ) were determined using a software. Subsequent to radiographic measurements, clinical measurements were carried out meticulously during flap procedures. Then linear measurements were carried out using a periodontal probe to determine the defect depth and its mesiodistal width. Then the amount of correlation between these two measurements was assessed by Pearson's correlation coefficient.

**Results:**

The correlation between clinical and radiographic measurements in defect depth determination, in the evaluation of defect angle and in determination of defect width were 88%, 98% and 90%, respectively.

**Conclusions:**

Indirect digital radiographic technique can be used to diagnose intra-osseous defects, providing a better opportunity to treat bony defects.

## 1. Background

In periodontal diseases, the bone destruction pattern is divided into horizontal (even) and oblique (vertical/angular) defects. In the vertical pattern, bone destruction does not proceed in a symmetrical pattern. The severity of bone destruction varies in different parts around the tooth, which explains why the alveolar crest does not correspond to cemento-enamel junction (CEJ) and is not parallel to it (1). This bone destruction pattern gives rise to bony defects in which the base of the defect is located more apical to the alveolar crest (2). Diagnosis and accuracy in determining the exact location, extent and configuration of bony defects are of utmost importance to determine prognosis, to plan treatment and to preserve the teeth in the long run (3). Because determination of the depth and to some extent, the width of bony defects is an important parameter in the prognosis of treatment, it is important to accurately measure these two parameters on radiographs to develop a correct and appropriate treatment plan (4). Recently, digital radiography has attracted a lot of attention in determining the depth, width and topography of bony defects and progression of the defect since loss of bone density and height should be evaluated using an automated instrument to diagnose periodontal lesions and assess the treatment success (4). There are only a limited number of studies which have evaluated radiographic views of bony periodontal defects with inconclusive results (5). Pepelassi et al. (2000) evaluated the potential of conventional radiographic techniques in the diagnosis of intra-osseous periodontal defects in comparison with intra-operative evaluations and concluded that:

1. Radiographic techniques have limited capacity to determine and diagnose bony defects.

2. The accuracy of PA radiographs depends on the number of wall defects, the depth and bucco-lingual width and the location in the jaws.

3. It is difficult to characterize small and shallow defects on radiographs (6).

Kelin et al. evaluated the depth and width of bony defects as a diagnostic factor and changes in defect width as a determinant of periodontal healing in intra-osseous defects treated with GTR 6 and 24 months after surgery. In that study, subjects with intra-osseous defects were selected and treated with ePTFE (expanded polytetrafluoroethylene, a non-absorbable membrane). A computer was used to determine the depth, width and angulations of the defect. The bonefill had been preserved 24 months after surgery. They also concluded that the depth of the intra-osseous component is a more appropriate diagnostic parameter compared to the angulations of the defect (4).

## 2. Objectives

The aim of the present study was to evaluate the diagnostic value of indirect digital radiography (with the parallel technique) in determining the topography of periodontal bony defects and compare the results with real measurements.

## 3. Patients and Methods

### 3.1. Patient Selection

In this cross-sectional study, 20 interproximal bony defects were selected in patients who had undergone phase one periodontal therapy and were candidates for periodontal surgery. The inclusion criteria included no contraindications for periodontal surgery and exposure to X-rays. Patients with shallow palate or elevated floor of the mouth were excluded. Defects in the 5↔5 area were preferably selected. Pre-operative periapical radiographs were provided using the parallel technique in an XCP film holder (Dentsply, Rinn). One radiograph was provided for each interproximal defect. In order to determine the vertical and horizontal difference between the central ray and orthoradial projection, two pieces of orthodontic wire with a specific length were placed at a premeasured distance from each other on the mandibular side of the film holder. Then horizontal magnification was calculated by dividing the wire length on the radiograph by its real length. Vertical magnification was calculated by dividing the distance between the wires on the radiograph by their real distance. The radiographic machine Philips Oralix 655 and Kodak E-speed intra-oral films (Eastmary Kodak) were used in the radiography procedure (56 kVp, 7.5 mA). The radiographs were transferred to a computer using Mustek P3600 A3 PRO Scanner with a resolution of 400 pixels. Imag J (ver. 1.34) software (National Institutes of Health) was used for measurements on a computer.

### 3.2. Radiographic Examination

**Figure 1:**
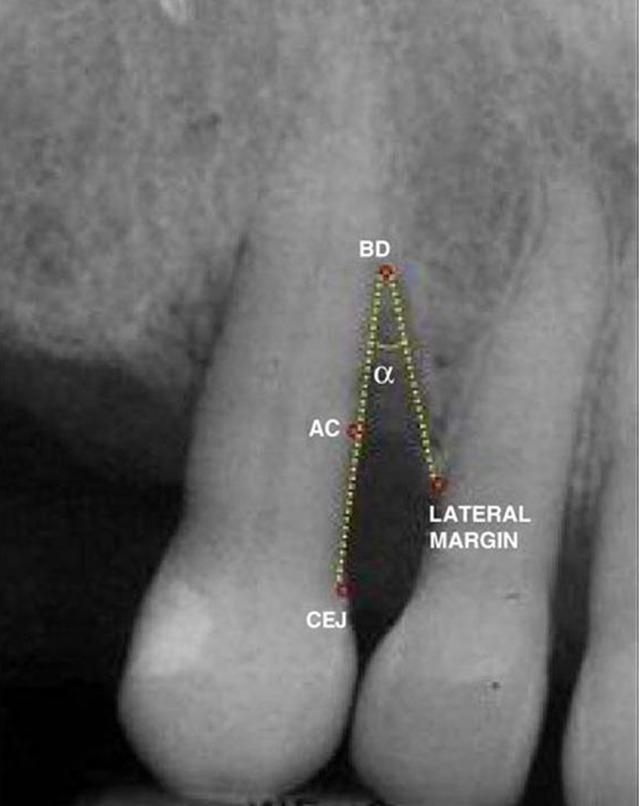
Radiographic measurements

Linear Measurement T software was used to open the radiographic images and in a manner similar to Photoshop software the following parameters were determined (Figure 1). BD is the most coronal point where periodontal ligament (PDL) continuity is observed. If PDL could not be determined, the point at which the alveolar crest (AC) projection contacted the root surface was selected as the landmark. If both landmarks were present, the first one was designated as BD and the second was designated as AC. If multiple bone contours were visible, the most apical contour contacting the root surface was designated as BD and the most coronal one was designated as AC. CEJ was the fixed coronal reference for these measurements. After CEJ, AC and BD were determined on the screen, the following measurements were carried out:

Defect depth = CIJ/BD - CEJ/AC

Defect width: Defect width is the distance between the mesial and distal borders of the bony defect, which is the distance between the lateral margin of the defect and the AC on the root surface.

Defect angle: Defect angle is the angle between the lateral defect wall and the root surface, which is drawn in the following manner:

One wall of the angle extends from CEJ to BD and the other wall extends from BD to the lateral margin of the defect. Radiographic measurements are made after drawing the above-mentioned lines. Subsequent to radiographic measurements, clinical measurements are carried out accurately during flap elevation. Measurements are made after removal of the granulation tissue and before only resective or regenerative surgical treatment. Bony defects not located on proximal surfaces were excluded from the study. At first, vertical defect type (the number of remaining walls) was determined and the following measurements were carried out:

Liner measurement: Linear measurement was carried out using a calibrated periodontal probe (PCPUNC-15, Hufriedy) to the nearest 0.5 mm:

1. Depth of the osseous defect: the distance between the alveolar crest and the base of the osseous defect.

2. Mesiodistal width of the osseous defect: the distance between the root surface and the osseous border of the defect in a mesiodistal direction at the level of the bony crest or the distance between the mesial and distal borders of the osseous defect. Accordingly, the minimum dimensions for a defect are: width = 0.5 mm; depth = 0.5 mm.

3. Defect angle: A hydrosol (elastomeric polyvinylsiloxane impression material) impression was taken from the defect.

This impression material was injected into the defect after mixing. Caution was exercised not to introduce bubbles into the material. The material was set after 6-7 minutes and then a sharp blade was used to draw an imaginary plane which extended from the alveolar crest to the buccal (or lingual) plate of the proximal tooth. Subsequently, the impression was removed from the defect using an explorer; then, the tips of the two forks of a pair of calipers were placed tangential with the two proximal walls of the impression and fixed. Then, the angle between the lateral wall of the defect and the root surface was determined. In both digital and clinical measurements, a periodontist carried out the measurement procedures three times and the mean of the three measurements was reported as the final value. This technique minimized intrarater error. After clinical and radiographic measurements were completed, the results were compared to evaluate similarities and differences between the methods. These were tested with Pearson’s correlation coefficient. All the statistical analyses were performed using SPSS version 15. A P-value under 0.05 was considered statistically significant. 

## 4. Results

### 4.1. Correlation Between Clinical and Radiographic Measurements in Determining the Defect Depth

**Figure 2:**
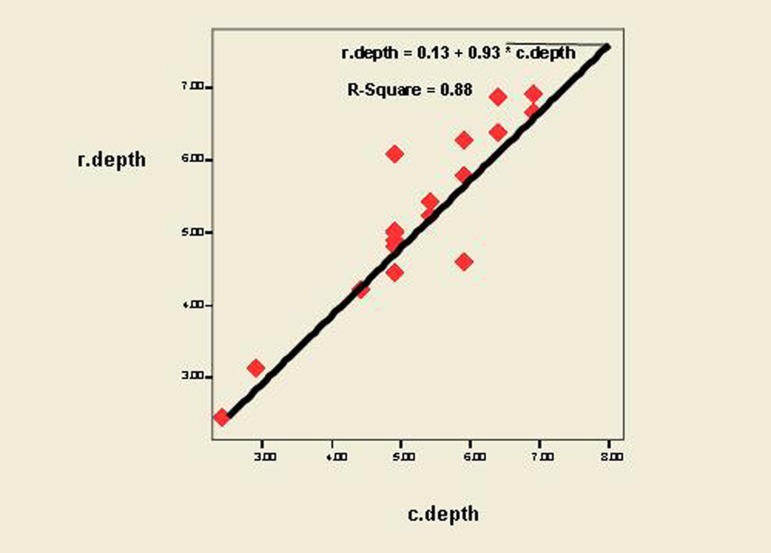
Correlation and regression between clinical and radiographic measurements in determining the defect depth

According to (Figure 2) and Pearson’s correlation coefficient, the correlation between radiographic and clinical measurements in determining the defect depth was strong (r-square = 0.88, P < 0.001).

### 4.2. Correlation Between Clinical and Radiographic Measurements in Determining the Defect Angle

**Figure 3:**
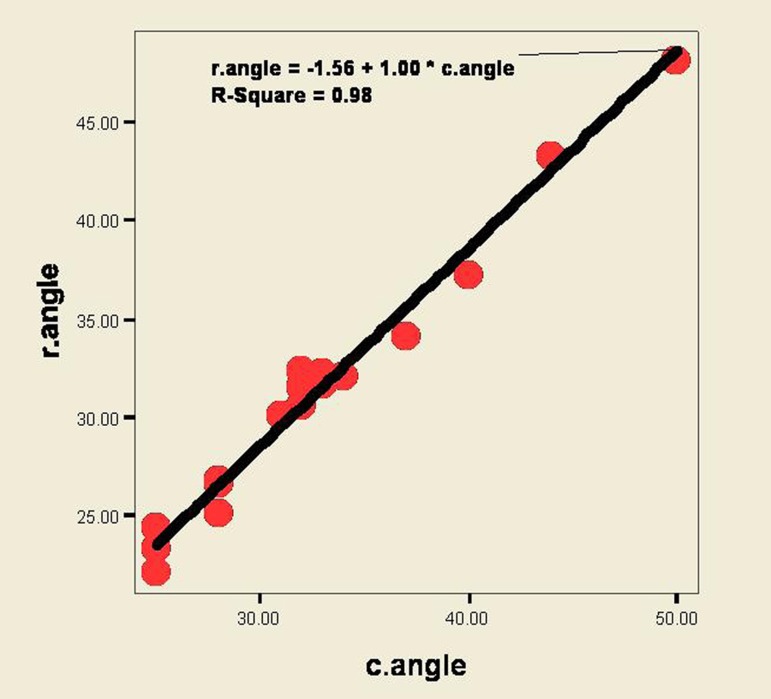
Correlation and regression between clinical and radiographic measurements in determining the defect angle

According to (Figure 3) and Pearson’s correlation coefficient, the correlation between radiographic and clinical measurements in determining defect angle was strong (r-square = 0.98, P < 0.001), demonstrating a strong correlation according to regression equation.

### 4.3. Correlation Between Clinical and Radiographic Measurements in Determining the Defect Width

**Figure 4:**
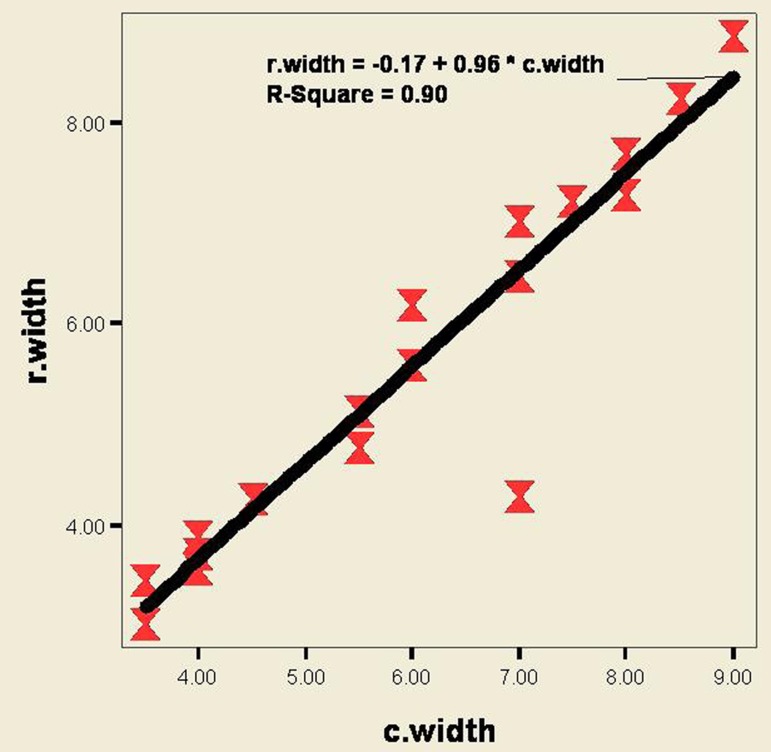
Correlation and regression between clinical and radiographic measurements in determining the defect width

According to (Figure 4) and Pearson’s correlation coefficient, agreement between clinical and radiographic measurements in determining defect width was strong (r-square = 0.90, P < 0.001).

### 4.4. Correlation Between Clinical and Radiographic Measurements in Determining the Defect Depth, Width and Angle

**Figure 5:**
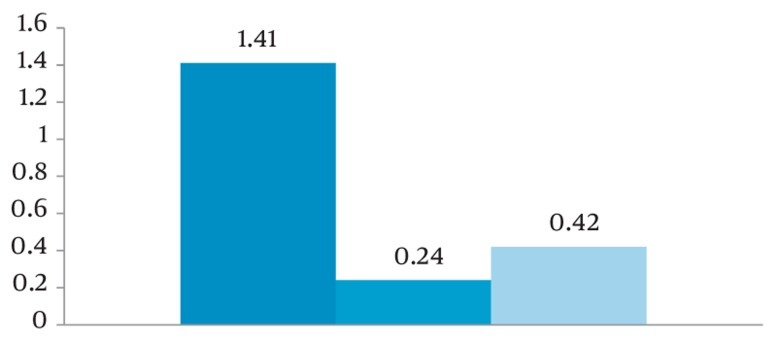
Mean differences between clinical and radiographic measurements of the defect angle, depth and width

According to (Figure 5), the mean differences between clinical and radiographic measurements of defect angle, depth and width were 1.41, 0.24 and 0.42, respectively.

## 5. Discussion

The results of the present study showed that digitized parallel periapical radiographs have a high level of correlation with clinical measurements. Therefore, the technique can be used to determine prognosis and also for treatment planning in osseous defects of the jaw. In the present study, the differences between radiographic and clinical measurements in relation to defect width and depth were 0.42 and 0.24, respectively, with no statistically significant differences; i.e. radiographic measurement was similar to clinical measurement. The results of the present study regarding width and depth measurements were respectively, consistent and inconsistent with the results of a study carried out by Pepelassi et al. (6) which compared the potential of conventional periapical and panoramic radiographic techniques in the determination and imaging accuracy of intra-osseous lesions compared to evaluations during surgical procedures. They evaluated the presence and measured the dimensions of osseous lesions during flap surgery, measured the distance between the alveolar crest and the lowermost part of the lesion (BD) on radiographs and measured the mesiodistal width of the lesion clinically using a periodontal probe. The results of their study showed that periapical radiographs were three times more efficient than panoramic views in determining the presence of osseous lesions. The results also revealed that the depth of the lesion by the two mentioned radiographic techniques is depicted bigger than the actual lesion size. This controversy might be attributed to the location, the types of teeth and jaw and the greater depth of the defects in the study carried out by Pepelassi et al. The results of the present study in this respect are consistent with those of Kelin et al. (4). They evaluated the depth and width of the lesion as a factor involved in determining prognosis and changes in the width of the lesions as a factor involved in demonstrating repair of osseous lesions treated with GTR, 6 and 24 months after surgery. Periapical radio graphs were provided and CEJ-AC and CEJ-BD distances were measured at baseline and 6 and 24 months after surgery using digitized CCD camera and a computer. The results of the study demonstrated statistically significant differences in bone filling and bridging of wall defects, 6 and 24 months after surgery compared to conventional treatment modalities. The authors concluded that the radiographic analysis used in the study is a precise method for the evaluation of intra-osseous lesions and treatment results and measurements are close to actual dimensions. In a study carried out by Wolf et al. (7), selected digital modifications (filters, scatter, structure) in radiographic images did not result in more reproducible or more valid results for measuring bone resorption in interproximal lesions compared with un-modified digital radiographs. Although the radiographic values were lower than the clinical values in the present study, the differences were not statistically significant, with a strong correlation between radiographic and clinical values. Periodontal diseases result in endosteal resorption, which produces radiolucencies; however, bone resorption does not produce a uniformly recognizable view. Extra- and intra-oral radiographic techniques are used to record changes. Radiographs are two-dimensional representations of a three-dimensional structure. Therefore, the image is not an accurate one and bone resorption on the radiograph is depicted smaller than the actual lesion size. Therefore, measurements of bone resorption on radiographs are not accurate to diagnose osseous lesions. In 1980, Goodson et al. used a computer as an aid in the linear evaluation of bone resorption (8). Given the technological advances and introduction of digital radiographic technique, it has extensively been used in medicine and its use in dentistry is rapidly on the rise. Digital radiographic technique is carried out in direct and indirect procedures. The main advantage of digitized radiographs is the ability to manipulate and enhance the quality of images using various software programs (5). In most cases, enhancement, which is carried out by a modified contrast, optimized illuminosity and decreased image noise, results in a visually-optimized image. In fact, image enhancement produces an optimized version of the primary image (9). However, a visually-optimized image does not necessarily result in better interpretation of the image. Manipulation of a digital image does not increase data; it only makes the structures better visible by condensing the data available. In such cases the possible decrease in the data available is a consideration. This higher coordination is suitable for low-quality images which have low contrast, or are overexposed or underexposed. Various software programs are available for the manipulation and linear evaluation of digitized radiographs (10). Various studies using different software programs have yielded differing and sometimes contradictory results. In the present study, linear evaluation during surgery and also on conventional periapical radiographs were carried out on 0.5-mm intervals; however, linear evaluations on digitized radiographs were carried out by manipulation on a computer at 0.01-mm intervals, which might be the main reason for the higher value of digitized radiographs compared to conventional periapical radiographs. One of the limitations of the present study was the number of samples and the absence of evaluation of the above-mentioned method in determining the topography of various interproximal osseous lesions (one-wall, two-wall and three-wall defects). Therefore, it is suggested that further studies should be carried out with larger sample sizes for evaluating various types of periodontal osseous defects.

The results indicate that digital enhancement can result in an increased ability to diagnose intra-osseous defects, paving the way for a more appropriate decision-making process to treat intra-osseous lesions more properly.
